# Potential chemokine biomarkers associated with PTSD onset, risk and resilience as well as stress responses in US military service members

**DOI:** 10.1038/s41398-020-0693-1

**Published:** 2020-01-23

**Authors:** Lei Zhang, Xian-Zhang Hu, Xiaoxia Li, Ze Chen, David M. Benedek, Carol S. Fullerton, Gary Wynn, Biomarker team, James A. Naifeh, Hongyan Wu, Natasha Benfer, Tsz Hin H. Ng, Poblo Aliaga, Hieu Dinh, Tzu-Cheg Kao, Robert J. Ursano

**Affiliations:** 1 Center for the Study of Traumatic Stress, Department of Psychiatry, Uniformed Services University of the Health Sciences, Bethesda, MD 20814 USA; 2 Department of Preventive Medicine and Biostatistics, Uniformed Services University of the Health Sciences, Bethesda, MD USA

**Keywords:** Neuroscience, Molecular neuroscience

## Abstract

Cytokines, including chemokines, are small secreted proteins, which specifically effect on the interactions and communications between cells. Pro-inflammatory cytokines are produced predominantly by activated macrophages and are involved in the upregulation of inflammatory reactions. Dysregulation of cytokines is associated with post-traumatic stress disorder (PTSD). Here, we use both before-and-after and case–control studies to search for potential chemokine biomarkers associated with PTSD onset, risk, and resilience as well as stress responses in US military service members deployed to Iraq and Afghanistan. Blood samples and scores of the PTSD Checklist (PCL) were obtained from soldiers pre- and post deployment (pre, post). Forty chemokines were measured using the Bio-Plex Pro Human Chemokine Panel Assays. The before-and-after analysis showed potential markers (CCL2, CCL15, CCL22, CCL25, CXCL2, and CXCL12) are associated with PTSD onset, and CCL3, CXCL11, and CXCL16 are related to stress response. The case–control study demonstrated that CCL13, CCL20, and CXCL6 were possible PTSD risk markers, and CX3CL1 might be a resilience marker. In addition, CCL11, CCL13, CCL20, and CCL25 were correlated with the PCL scores, indicating their association with PTSD symptom severity. Our data, for the first time, suggest that these dysregulated chemokines may serve as biomarkers for PTSD onset, risk, and resilience as well as stress responses, and may benefit developing approaches not only for PTSD diagnosis but also for PTSD treatment.

## Introduction

Post-traumatic stress disorder (PTSD) is a debilitating mental disorder with prevalence rates of >7% and 12% in the US civilians^1^ and military, respectively^2^. Due to the Iraq and Afghanistan Wars, the rate of PTSD is significantly increased in the combat veteran population^3^. About 10–18% of combat troops who served in Operation Enduring Freedom or Operation Iraqi Freedom are likely to suffer from PTSD^4^, who also experience dysregulation of the immune system^5–10^. Increased rates of rheumatism, sarcoidosis, multiple sclerosis, and other diseases associated with the immune system are reported in veterans coming back from the Gulf War^10^.

Cytokines, including chemokines, interferons (INF), interleukins (IL), lymphokines, and tumor necrosis factors (TNF), have been associated with PTSD^11–13^. Individuals with PTSD have higher levels of peripheral cytokines (IL-2, IL-4, IL-6, IL-8, IL-10, and TNF-α) than age- and gender-matched healthy controls^12^, suggesting a generalized inflammatory state in PTSD patients. However, studies of cytokines in PTSD have yielded mixed results. While higher levels of IL-1β^14,15^, IL-6^16^, and TNF-α^17^ are found in the plasma^18^ of PTSD patients compared with control subjects, no significant differences in the levels of IL-1β^17^, IL-6^17,19^, or IL-8^19^ are observed in PTSD. These inconsistent findings may partly be explained by variations in sample characteristics, such as differences in the type of trauma experienced (e.g., child vs adult trauma), duration of the trauma exposure (e.g., studies right after traumatic events or after an ongoing threat, such as war), civilian vs military populations, location (geographical differences), medications (patients with or without medication), and assay procedures (ELISA vs Western Blot, several vs single cytokine assay, and saliva vs blood). Here, we search for potential markers predicting PTSD onset, risk, and resilience as well as stress response using a before-and-after study and a case–control study examining the blood chemokine levels among samples from US Army soldiers in the Iraq and Afghanistan wars.

Based on previous findings showing that PTSD is deployment-related, we hypothesized that soldiers may respond to deployment as a traumatic life event. The differences of blood cytokine levels between subjects without PTSD before deployment (case–control pre) and the same subjects who became PTSD after deployment (case post) served as disease markers; differences of basal levels of blood cytokine between control pre (without PTSD) and case–control pre may be the risk markers for PTSD; difference of blood cytokine levels between control pre (without PTSD) and control post (without PTSD) may be the stress response markers; and the differences between case post (with PTSD) and control post (without PTSD) may be markers for resilience.

## Methods

### Subjects

This project was designed as both before-and-after and case–control studies to examine changes in blood cytokine prior to and after development. Participants were Guamanian National Guard Soldier (NGS) volunteers with or without PTSD recruited from April of 2013 to August of 2014. The NGS were deployed to Afghanistan in support of US combat operations. They were voluntarily in an anonymous self-reported behavioral health survey 3 months (April 2013) prior to their deployment (pre) to Iraq, and then again 3 months after their deployment (post, August 2014). Of the original 526 soldiers, 276 of the soldiers were available for follow-up at the post-deployment time point. Of those 276 soldiers, 249 were able to be matched with their pre-deployment time point due to the blinding process. Of those 249 soldiers, 25 (10%) were diagnosed with PTSD at the post-deployment time point. A number of blood samples from PTSD patients were lost, leaving 13 PTSD samples available. Age- and gender-matched pre-deployment and post-deployment healthy controls were recruited (*n* = 26), but only 23 had available blood samples. Matched subjects with available blood samples were divided into four groups: control pre (without PTSD before deployment, *n* = 23), case–control pre (without PTSD before deployment, *n* = 13), control post (without PTSD after deployment, *n* = 23), and case post (with PTSD after deployment, *n* = 13).

### Assessments of demographic information and PCL

PTSD symptoms were assessed using the PCL, a 17-item, DSM-based, self-report measure with well-established validity and reliability^20^. Current PTSD was determined based on endorsement of DSM-IV criteria, and a PCL total score ≥ 50. PTSD symptom severity was determined using the PCL total score. Demographic data, such as age, gender, and race, were collected. Controls (non-PTSD) were age-, race-, deployment time-, and sex-matched subjects. All study procedures were approved by the Institutional Review Board at the Uniformed Service University (USUHS), and all participants were given written informed consent.

### Blood sample collection and chemokine analysis

Venous blood (8.5 mL) was collected with BD vacutainer glass whole-blood tubes (Product #: 364606. BD, New Jersey). The whole-blood samples were centrifuged at 1300 × *g* for 10 min at 4 °C. Then the plasma was transferred to a labeled fresh Eppendorf tube and stored at −80 °C. Chemokine levels in the plasma were quantified using the Bio-Plex Pro Human Chemokine Panel Assays (Bio-rad, Bio-Rad Laboratories, Hercules, CA, USA). A luminex assay uses a bead-based, flow cytometric platform dedicated to multiplex analysis. Similar to ELISA, a majority of assays are designed according to a capture sandwich immunoassay format. Briefly, the capture chemokine antibody-coupled beads are first incubated with antigen standard samples. The plate is then washed to remove any unbound materials, followed by incubation with biotinylated detection antibodies. After washing away the unbound biotinylated antibodies, the beads are incubated with a reporter streptavidin–phycoerythrin conjugate (SA-PE). Following removal of excess SA-PE, the beads are passed through the array reader, which measures the fluorescence of the bound SA-PE. Samples were prepared according to the manufacturer’s instructions. Absolute chemokine levels were calculated based on the mean fluorescence intensity of chemokine standard curve. The Bio-Plex Pro Human Chemokine Panel Assays allow the detection and quantification of the following 40 chemokines from human biological samples (Table 1).

**Table 1:** (a) The results from testing of 40 human chemokines; (b) summary of potential chemokine marker identification.

(a)			
6Ckine/CCL21	Gro-α/CXCL1	IL-16	MIP-1α/CCL3
BCA-1/CXCL13	Gro-β/CXCL2	IP-10/CXCL10	MIP-1δ/CCL15
CTACK/CCL27	I-309/CCL1	I-TAC/CXCL11	MIP-3α/CCL20
ENA-78/CXCL5	IFN-ϒ	MCP-1/CCL2	MIP-3β/CCL19
Eotaxin/CCL11	IL-1β	MCP-2/CCL8	MPIF-1/CCL23
Eotaxin-2/CCL24	IL-2	MCP-3/CCL7	SCYB16/CXCL16
Eotaxin-3/CCL26	IL-4	MCP-4/CCL13	SDF-1α + β/CXCL12
Fractalkine/CX3CL1	IL-6	MDC/CCL22	TARC/CCL17
GCP-2/CXCL6	IL-8/CXCL8	MIF	TECK/CCL25
GM-CSF	IL-10	MIG/CXCL9	TNF-α

(b)				
_Chemokine_	_CC_	_CXC_	_CX3C_	_C_
PTSD markers (case–control pre/case post)	CCL2 CCL15 CCL22 CCL23 CCL25	CXCL2 CXCL12	None	None
Stress markers (control pre/control post)	CCL3	CXCL5 CXCL11 CXCL16	None	None
PTSD risk markers (case–control pre/control pre)	CCL13 CCL20 CCL23	CXCL5 CXCL6	None	None
Resilience marker (case post/control post)	None	None	CX3CL1	None

### Statistical analyses

Group comparisons with respect to sociodemographic variables were performed using chi-square tests for the categorical data and *t* tests for the continuous data. *P*-values < 0.05 were considered statistically significant. Regression tests (SPSS Version 24) were used to examine the relationships between chemokine levels and severity of PTSD symptoms. Scatter plots were used to display the relationship between chemokines and the severity of PTSD symptoms. Based on the mean values and standard deviation of chemokine in different groups and given the significant at 0.05, the power in our sample size is >0.90.

## Results

### Significant differences of chemokine levels between case–control pre and case post

**Fig. 1:**
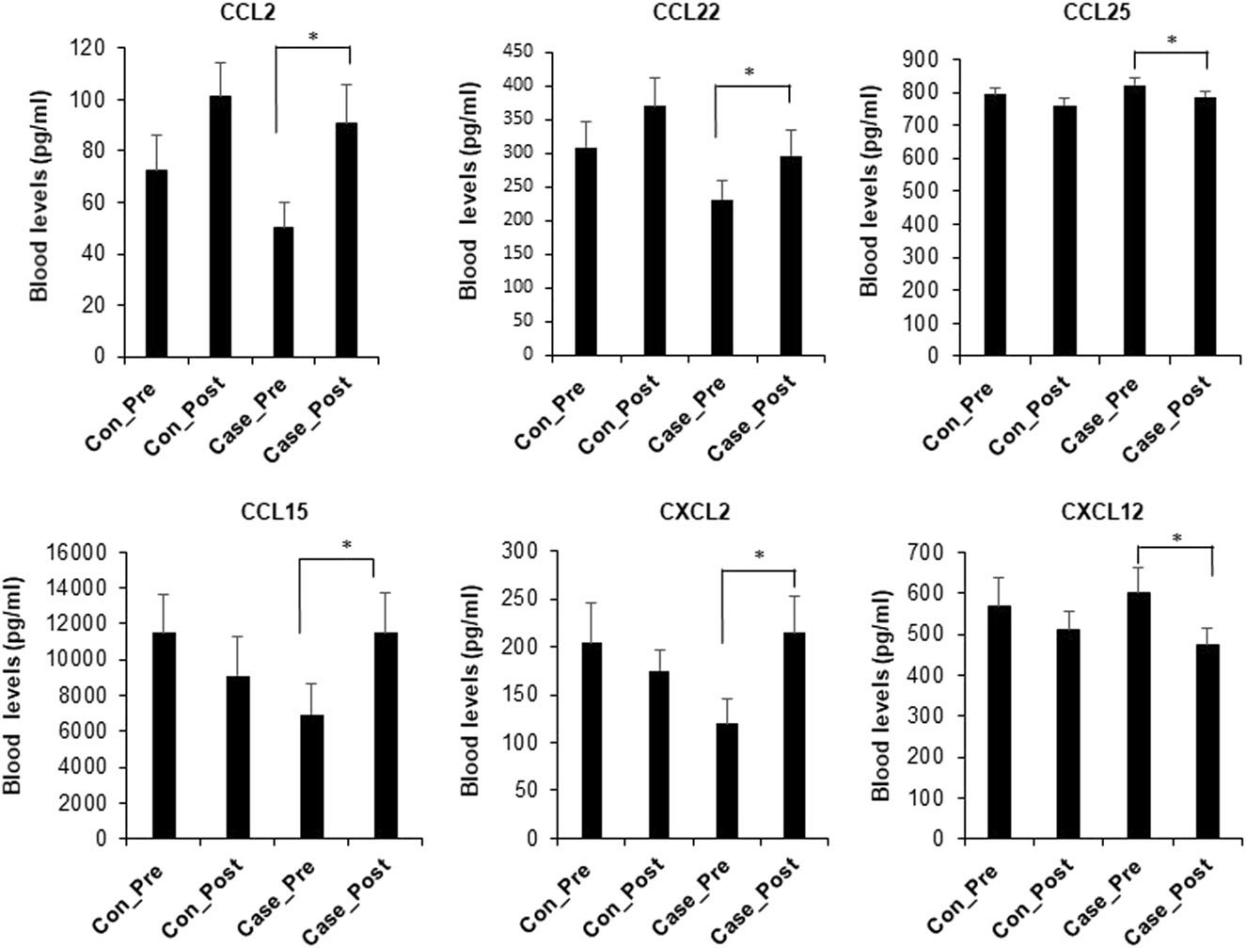
Differences of chemokines in case post vs case–control pre. The chemokine levels are measured in the plasma at pre and post. The bar graphs show the means ± SE for each of the tested groups: control pre (*n* = 23), control post (*n* = 23), case pre (case–control pre, *n* = 13), and case post (*n* = 13). *P < 0.05.

Figure 1 shows that there are six dysregulated chemokines in case post compared with case–control pre. Concentrations of CCL2, CCL22, CCL15, and CXCL2 were significantly increased in the case post compared with case–control pre (*P* < 0.05), while CCL25 and CXCL12 were significantly decreased in the case post vs case–control pre (*P* < 0.05).

### Significant differences of chemokine levels between control pre and control post

**Fig. 2:**
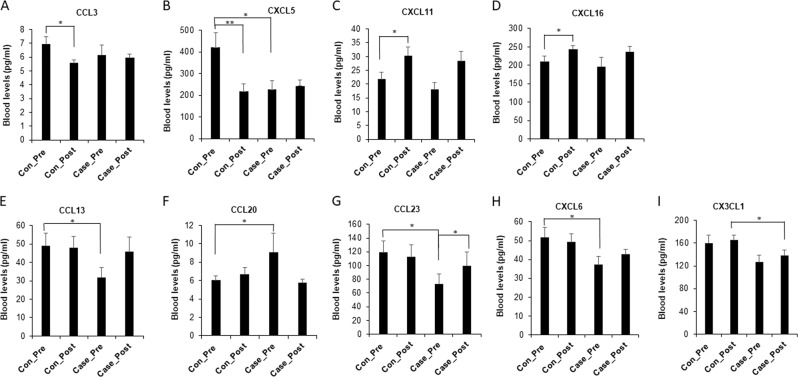
The comparisons of chemokines levels among tested groups. **a**–**d** Differences of chemokines in control post vs control pre. The chemokine levels were measured in the plasma at pre and post. The bar graphs show the means ± SE for each of the tested groups: control pre (*n* = 23), control post (*n* = 23), case pre (case–control pre, *n* = 13), and case post (*n* = 13). **e**–**h** Differences of chemokine levels between control pre and case–control pre. The chemokine levels were measured in the plasma at pre and post. The bar graphs show the means ± SE for each of the tested groups: control pre (*n* = 23), control post (*n* = 23), case pre (case–control pre, *n* = 13), and case post (*n* = 13). **i** Differences of chemokine CX3CL1 in case post vs other tested groups. The chemokine levels were measured in the plasma at pre and post. The bar graphs show the means ± SE for each of the tested groups: control pre (*n* = 23), control post (*n* = 23), case pre (case–control pre, *n* = 13) and case post (*n* = 13). *P < 0.05, **P < 0.01.

Figure 2a–d demonstrates that there are four dysregulated chemokines in control pre as compared with control post.

Concentrations of two chemokines (CXCL11 and CXCL16) were significantly increased in the control post compared with control pre (*P* < 0.05), while two chemokines (CCL3 and CXCL5) were significantly decreased in the control post vs control pre. Meanwhile, CXCL5 was also significantly lower in the case–control pre than in the control pre (*P* < 0.05).

### Significant differences of chemokine levels between control pre and case–control pre

Figure 2e–h reveals that there are four dysregulated chemokines in the control pre vs case–control pre. Concentrations of three chemokines (CCL13, CCL23, and CXCL6) were significantly lower in the case–control pre than in control pre (*P* < 0.05), while one chemokine (CCL20) was significantly higher in the case–control pre than in the control pre (*P* < 0.05). In addition, CCL23 was significantly higher in the case post than in the case–control pre (*P* < 0.05).

### Dysregulation of CX3CL1 in case post vs control post

Figure 2i shows that CX3CL1 was significantly decreased in the case post compared with control post, with no difference between control pre and control post.

### Figure 3 demonstrates all possible relationships of potential chemokine markers between PTSD and risk as well as PTSD and stress response

**Fig. 3:**
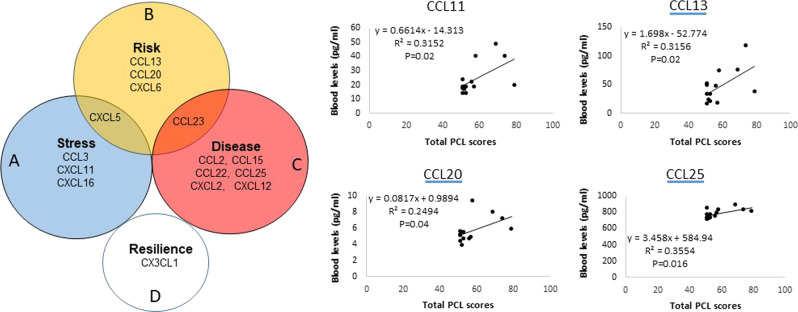
Venn diagram showing overlap of expression of potential chemokine markers for PTSD occurrence, risk, and resilience, as well as stress response. Four chemokines are associated with PTSD severity. PCL scores were used to determine the disease severity in PTSD subjects. A higher score represented more severe PTSD. Regression was used to analyze the correlation between chemokine levels and PCL scores. Each dot or diamond symbol represents the data from a subject with PTSD. Both correlation coefficient (R) and *P*-values were presented. Levels of CCL13, CCL23, CCL25, and CXCL11 are significantly associated with PCL scores.

Set A, in blue, represents dysfunction chemokines which might be risk markers for the stress response (Fig. 3). The yellow circle, set B, represents dysfunction chemokines, which might be markers for a future risk of PTSD. Set C, in red, represents dysfunction chemokines, which might serve as markers for current PTSD. Each separate type of marker is imagined as a point somewhere in the diagram. Two chemokines, CXCL5 and CCL23, are in A and B and B and C, respectively, corresponding to points in which the areas overlap. D is possible resilience markers, since it is deregulated in the case post compared with control post, and no difference between control pre and control post. Thus, it is possible that chemokines in one area only are sole markers for defied conditions (PTSD onset, risk, and resilience, and stress response).

### Four chemokines were associated with severity of PTSD symptoms

Figure 3 shows that levels of four chemokines in PTSD are significantly correlated with PCL scores.

## Discussion

In this study, we investigated plasma levels of 40 cytokines in samples from US NGS, both pre- and post deployment during the Iraq and Afghanistan wars. Using before-and-after and case–control study designs, a well-defined population (US NGS, one location), and a stressor (deployment to the war), we found that the significant alterations were in chemokines, which were potential markers for PTSD onset, risk, and resilience, as well as stress response, thus paving the way for treatment and preventive interventions (Table 2). We used a luminex assay, an effective tool in cytokine detection and quantification. A luminex assay is different from conventional technologies, such as ELISA, which measure individual cytokines^21^. It has the capacity to measure multiple different cytokines simultaneously in a single run of the assay with small sample-size requirements. Simultaneous measurement of multiple cytokines in the blood provides an experimental strategy to resolve complex interactions among signaling molecules^22^ to obtain an objective pattern of numerous cytokines within an experiment, which provides a more inclusive and comprehensive depiction of PTSD.

**Table 2:** Potential chemokine markers for PTSD onset, risk, and resilience, as well as stress response.

	control post (without PTSD) (n = 23)	case post (with PTSD (n = 13)	case-control pre (without PTSD) (n = 13)
control pre (without PTSD) (n = 23)	Stress response markers: A		Risk markers: B
case-control pre (without PTSD) (n = 13)		Disease markers: C	
control post (without PTSD) (n = 23)		Resilience markers: D	

We used a before-and-after research design to test relationships between deployment (war exposures) and PTSD onset or stress response. This method measured the effects of deployment on the blood levels of cytokines in PTSD and non-PTSD controls. Changes of cytokine levels were compared within the same participant pre- vs post deployment. The goal of the design is to examine if the deployment, PTSD, or stress response link has changed over time. In theory, this link would be due to the deployment. In addition, this prospective study followed a group of soldiers with deployment to the Afghanistan and Iraq wars up to a year. We found a heightened inflammatory state in PTSD patients when analyzing chemokine levels between case post (with PTSD) and case–control pre (without PTSD). A group of chemokines including five CCs (CCL2, CCL3, CCL15, CCL23, and CCL25) and two CXCs (CXCL2 and CXCL12) were dysregulated in the case post vs case–control pre (without PTSD). CCL2, CCL22, CCL15, and CXCL2 were significantly higher in case post than in case–control pre, while CCL25 and CXCL12 were significantly lower in case post than in case–control pre, indicating those up- and downregulated chemokines were potential markers for PTSD onset.

Castellani et al.^23^ highlight the significance of pro-inflammatory chemokine activation, such as CCL2 (MCP-1), in conditions of chronic stress. Hoge et al.^13^ show increased chemokine CCL2 level in PTSD patients versus controls. It is also found that CXCL12 (SDF-1) levels are significantly increased in PTSD versus control groups^24^. Several possible factors may contribute to the different results between ours and Onglide’s. First, subjects in this study are active duty military service members who are exposed to unique war-related traumatic events, while their subjects are civilians who may have experience with non-war-related traumatic events. Second, the PTSD symptoms and cytokines in this study are evaluated right after or during deployment, while Onglide’s variables may be determined years after traumatic events. In addition, the cytokines are examined using self-controlled or before-and-after design in our study, while the cytokines are examined using case–control in their study. Finally, the difference of disease stages between the two studies may also be a reason for this inconsistency.

Potential chemokine markers for stress response were identified when the chemokine levels were analyzed between the control pre vs control post (without PTSD). We demonstrated that CCL3 and CXCL5 were downregulated while CXCL 11 and CXCL16 were upregulated in the non-PTSD control subjects after deployment (Fig. 2).

We also took advantage in using the samples of cases and controls to determine the possible cytokine markers for PTSD risk and resilience. We compared the concentration of chemokines between control pre (solders without PTSD pre deployment) and case–control pre (case controls who did not experience PTSD pre deployment, but developed PTSD by post deployment). We found that CCL13, CCL 23, and CXL6 were lower in the case–control pre than in control pre, while CCL20 was significantly higher in the case–control pre than in the control pre, indicating they may be markers of risk for PTSD. There is an overlap of CCL23 dysregulation among the control, non-PTSD case control, and case. Difference of CCL23 levels was also observed between case–control pre and case post. Therefore, it may be a “sticky” marker, which is in subjects at PTSD risk or with PTSD onset.

To identify resilience markers, we compared the levels of chemokines of control post and case post and found that blood CX3CL1 levels were lower in case post than in control post. Our data indicated that CX3CL1 might be a “resilience” molecule. Resilience is a beneficial quality that allows the subjects come back at least as strong as before. Rather than letting difficulties or failure overcome us and drain our resolve, we would find a way to rise from our hardship. The higher levels of CX3CL1 in the resilience subjects further supported that it might be a molecule to reduce PTSD risks. However, there was no difference of CX3CL1 between control pre and control post. The data indicate that, although deployment tended to increase blood CX3CL1 levels in PTSD (case–control pre vs case post), it did not alter the levels of CX3CL1 in controls (control pre vs control post). The data indicate that CX3CL1 might predict who would most likely not develop signs of PTSD after exposure to a stressful situation.

Chemokines have been divided into several sub-groups dependent on their chemical structures. They have four cysteine residues in conserved locations that are key to forming their three-dimensional shape^25^. Typical chemokine proteins are produced as pro-peptides, beginning with a signal peptide of approximately 20 amino acids. Their first two cysteines are situated close together near the N-terminal end of the mature protein, with the third cysteine residing in the center of the molecule and the fourth close to the C-terminal end^26^. A loop of approximately ten amino acids follows the first two cysteines is the N-loop^26^. This is followed by a single-turn helix, called a 310-helix, three β-strands, and a C-terminal α-helix. These helices and strands are connected by turns called 30 s, 40 s, and 50 s loops; the third and fourth cysteines are located in the 30 s and 50 s loops^26^. Although chemokines were originally identified as having chemotactic functions on immune cells, recent evidence has begun to elucidate novel, brain-specific functions of these proteins of relevance to the mechanisms of psychiatric disorders^27^. As we demonstrated in Table 1, the four main subfamilies are CC, CXC, CX3C, and C^26^. The CC chemokine (or β-chemokine) proteins have two adjacent cysteines (amino acids), near their amino terminus^26^. The two N-terminal cysteines of CXC chemokines (or α-chemokines) are separated by one amino acid, represented in this name with an “X”^26^. The C chemokines (or γ chemokines) are unlike all other chemokines in that they has only two cysteines; one N-terminal cysteine and one cysteine downstream. The cytokine that has three amino acids between the two cysteines and is termed CX3C chemokine (or d-chemokines). Only one CX3C chemokine, named fractalkine (or CX3CL1), is discovered to date. CX3CL1 is secreted and tethered to the surface of the cell that expresses it, serving as both a chemoattractant and as an adhesion molecule^26^.

CX3CL1 (fractalkine) is a large chemokine protein of 373 amino acids and contains multiple domains. It is the only known member of the CX3C chemokine family^28^. The polypeptide structure of CX3CL1 differs from the typical structure of other chemokines. CX3CL1 potently chemoattracts T cells and monocytes, while the cell-bound chemokine promotes strong adhesion of leukocytes to activated endothelial cells, where it is primarily expressed^29^. CX3CL1 elicits its adhesive and migratory functions by interacting with the chemokine receptor CX3CR1^30^ and expresses throughout the brain, particularly in neural cells. Its receptor is known to be present on microglial cells in the hippocampus^31^ and is essential for microglial cell migration^32^. CX3CL1 is associated with spatial learning, which might be involved in the regulation of glutamate neurotransmission, indicating its role in the protective plasticity process of synaptic scaling^33^. To our knowledge, this is the first study to demonstrate that CX3CL1 is associated with PTSD and many be a resilience marker.

Each type of chemokines exert their biological effects by interacting with G protein-linked transmembrane receptors called chemokine receptors that are selectively found on the surfaces of their target cells^25^. We have demonstrated the distribution of dysregulated chemokines in all classes of chemokines and found there is no dysregulated chemokine in the C subfamily in all tested subjects (Table 1). In PTSD, there are five CC and two CXC dysregulated chemokines, one CC and three CXC chemokines in controls (control pre vs control post), three CC and two CXC in case–control pre vs control pre, and one CX3C in case post vs control post. The unique distribution of dysregulated chemokines in different subfamilies provides first-hand information, which may help to develop a custom designed diagnostic tool for PTSD. After further relationship analysis (Fig. 3), we also found the overlap of expression of potential chemokines among the tested group (Fig. 3). Excusing CXCL5 and CCL23 from the dysregulated chemokine may leave makers for PTSD onset, risk, and resilience, as well as stress response, encouraging further investigation of the mechanisms of dysregulated chemokines in PTSD and stress studies.

Now, it is known that certain chemokines are associated with the neurobiological processes, including neurogenesis, modulation of the neuroinflammatory response, regulation of the hypothalamus–pituitary–adrenal axis, and modulation of neurotransmitter systems^13^. There are mechanisms shared across disorders^34^. For example, it is found that dysregulation of CCL11 is associated with impairment of hippocampal function in aging^35^—a distinct relevance to Alzheimer’s disease and depression in the elderly. In this study, we found that pro-inflammatory chemokines, such as CCL2, CCL12, and CCL13, which drive chemotaxis of pro-inflammatory cells to the inflamed or injured CNS^36^ were dysregulated in PTSD. We also demonstrated that CX3CL, which promotes glial cell activation and recruitment of CD4^+^ T cells into the CNS during neuroinflammatory processes^13^, is a potential resilience marker of PTSD.

We examined the relationships between dysregulated chemokines and PTSD severity and found four chemokines (CCL13, CCL23, CCL25, and CXCL11) were correlated with PCL scores (Fig. 3), indicating that they were associated with PTSD severity. To our knowledge, this is the first study to demonstrate that PTSD severity is associated, specifically, with four dysregulated chemokines. Here, we briefly discuss the information about those four chemokines in general. Alteration of CCL13 expression is particularly interesting because its gene is located on human chromosome 17 within a large cluster of other CC chemokines^37^. It binds to a cell surface G protein, which links chemokine receptors, such as CCR2, CCR3, and CCR5^38^ in monocytes, eosinophils, T lymphocytes, and basophils. It is involved in the regulation of allergic reactions such as asthma^39^. In the CNS, it drives chemotaxis of pro-inflammatory cells to the inflamed or injured sites. CCL13 can be induced by the inflammatory cytokines interleukin-1 and TNF-α.

The second PTSD severity-associated chemokine, CCL23, is a member of the CC subfamily, displays chemotactic activity on resting T lymphocytes and monocytes, and demonstrates no activity on activated T lymphocytes. It is slightly chemotactic for neutrophils and is a potent agonist of the chemokine (C–C motif) receptor 1. CCL23 predominantly expresses in the lung, liver tissues, bone marrow, and placenta^40^. Its receptor is CCR1 on peripheral blood lymphocytes and monocytes^41^.

The third chemokine associated with PTSD severity CCL25 belongs to the CC chemokine family. CCL25 is highly expressed in peripheral blood leukocytes, the pancreas and liver, with moderate levels in the thymus, spleen, and lung, and low expression levels in the small intestine, placenta, and prostate^42^. CCL25 binds to the chemokine receptor CCR9^43,44^ and plays a role in the development of T cells^45^. It is also chemotactic for thymocytes, macrophages, and dendritic cells. Finally, CXCL11, severity-associated chemokine, expresses in peripheral blood leukocytes. Although CXCL11 can be upregulated by IFN-γ^42^ and IFN-β^42^, and weakly induced by IFN-α^46^. We found there was no significant deference of IFN levels between PTSD group and non-PTSD controls.

Although this research has been done in well-controlled military population, we are still aware of its limitations and shortcomings. First, power analysis of sample size was >0.90. However, the sample size in this study appears to be small. Second, since it is not clinical trial, there is no intervention involved. It is apparent that the effects of intervention on inflammatory biomarkers needs to be studied using a large sample size and in an intervention situation.

In conclusion, dysregulated chemokines may represent strong candidate biomarkers, which can predicate PTSD onset, risk, and resilience, as well as stress responses. Searching for PTSD-associated chemokines may benefit developing approaches not only for PTSD diagnosis but also for PTSD treatment.
